# Effect of successive tuberculin skin test readings on healthcare professionals’ proficiency: an operational observational study in five high-tuberculosis-burden Brazilian municipalities, 2023-2024

**DOI:** 10.1590/S2237-96222025v34e20240795.en

**Published:** 2025-10-31

**Authors:** Dinah Carvalho Cordeiro, Elisa Barroso de Aguiar, Ronir Raggio Luiz, Daniel Souza Sacramento, Priscilla Lúcia Wolter Paolino, Maria Jacirema Ferreira Gonçalves, Anete Trajman

**Affiliations:** 1 Universidade Federal do Rio de Janeiro, Programa de Pós-Graduação em Clínica Médica, Rio de Janeiro, RJ, Brazil; 2 Universidade Federal do Rio de Janeiro, Instituto de Estudos em Saúde Coletiva, Rio de Janeiro, RJ, Brazil; 3 Secretaria Municipal de Saúde de Manaus, Departamento de Vigilância Epidemiológica, Ambiental, Zoonoses e da Saúde do Trabalhador, Manaus, AM, Brazil; 4 Rede Brasileira de Pesquisa em Tuberculose, Rio de Janeiro, RJ, Brazil; 5 Universidade Federal do Amazonas, Programa de Pós-Graduação em Enfermagem, Manaus, AM, Brazil

**Keywords:** Latent Infection, Training Courses, Tuberculin Test, Health Services, Observational Study, Infección Latente, Cursos de Capacitación, Prueba de Tuberculina, Servicios de Salud, Estudio Observacional

## Abstract

**Objective:**

To verify the effect of repeated readings of the tuberculin skin test on the proficiency of healthcare professionals.

**Methods:**

This was an operational observational study that compared the readings of professionals and lead trainers during training sessions conducted by the Ministry of Health in five Brazilian municipalities. The mean difference between readings, agreement, sensitivity, and specificity were calculated, considering the lead trainer as the gold standard for each of the first 50 readings. The Kappa index assessed interobserver agreement. The database originated from a training program on tuberculin skin test reading. The Statistical Package for the Social Sciences Statistics (SPSS) software was used for descriptive analyses.

**Results:**

168 professionals were trained, and a total of 5,929 readings were performed. The average number of readings per professional was 35.3±29.2 (minimum=5, maximum=122). The mean difference between the readings of the professionals and those of the lead trainers was 0.01 mm (±0.70 mm). The agreement was 93.0% for readings between 0 mm and 4 mm, 74.8% for readings between 5 mm and 9 mm, and 88.2% for readings of 10 mm or more (weighted Kappa coefficient 0.864). Sensitivity and specificity were 96.5% and 93.0% for the 5 mm cutoff point (the most commonly used in Brazil) and 88.2% and 95.0% for the 10 mm cutoff. None of the evaluated parameters changed during the first 50 readings.

**Conclusion:**

There was no effect of successive readings on the professionals’ proficiency, which was high from the first reading according to all parameters. These findings support the newly published training recommendations of the Ministry of Health.

## Resumen

**Objetivo:**

Verificar el efecto de lecturas sucesivas de la prueba de tuberculina en la aptitud de los profesionales de salud.

**Métodos:**

Se realizó un estudio observacional operacional que comparó las lecturas de profesionales y agentes multiplicadores en capacitaciones conducidas por el Ministerio de Salud en cinco municipios brasileños. Se calculó la diferencia media entre las lecturas, la concordancia, la sensibilidad y la especificidad considerando al agente multiplicador como estándar de referencia en cada una de las primeras 50 lecturas. La concordancia interobservador se evaluó mediante el índice Kappa. La base de datos provino de un programa de capacitación en lectura de la prueba de tuberculina. Se utilizó el software *Statistical Package* for the Social Sciences (SPSS) para los análisis descriptivos.

**Resultados:**

Se capacitaron 168 profesionales y se realizaron 5.929 lecturas. El número medio de lecturas por profesional fue de 35,3±29,2 (mínimo=5, máximo=122). La diferencia media entre las lecturas de los profesionales y las de los agentes multiplicadores fue 0,01 mm (±0,70 mm). La concordancia fue del 93,0% para lecturas entre 0 mm y 4 mm, 74,8% para lecturas entre 5 mm y 9 mm y 88,2% para lecturas de 10 mm o más (Kappa ponderado 0,864). La sensibilidad y especificidad fueron 96,5% y 93,0% para el punto de corte de 5 mm (el más usado en Brasil) y 88,2% y 95,0% para 10 mm. Ninguno de los parámetros evaluados sufrió alteración a lo largo de las primeras 50 lecturas.

**Conclusión:**

No hubo efecto de lecturas sucesivas en la habilidad de los profesionales, que fue elevada desde la primera lectura según todos los parámetros. Estos hallazgos respaldan las nuevas recomendaciones de capacitación del Ministerio de Salud recientemente publicadas.

**Ethical aspects**

This research respected ethical principles, having obtained the following approval data:

Research Ethics Committee: Universidade Federal do Rio de Janeiro

Opinion number: 5,936,814

Approval date: 10/3/2023

Certificate of Submission for Ethical Appraisal: 67116923.3.0000.5257

Informed Consent Form: Exempt.

## Introduction 

Caused by the infectious agent *Mycobacterium tuberculosis*, tuberculosis predominantly affects the lungs and is transmitted through the air. Transmission requires close and prolonged contact and occurs when individuals with untreated pulmonary tuberculosis release aerosolized bacilli by coughing, sneezing, singing, or speaking (1).

In 2023, more than 10 million people fell ill with tuberculosis worldwide, with the disease resuming its position as the leading cause of death by a single infectious agent that year (2). 

In 2011, it was estimated that 23.0% of the world’s population would be infected by *Mycobacterium tuberculosis* (3). In this condition, the bacterium remains viable in the body in an inactive form, without causing symptoms and without posing a risk of transmission. However, approximately 5.0% to 10.0% of these individuals may develop the disease throughout life, while others will be able to eliminate the infection naturally (4). Most of the new tuberculosis diagnoses come from this contingent of almost 2 billion people with the infection (1). 

After the initial infection, the risk of progression to tuberculosis is influenced by several factors, including the individual’s immune status and any associated health conditions. Classic symptoms include persistent cough, chest pain, fatigue, weight loss, fever, and night sweats (1).

The preventive treatment of tuberculosis is the primary intervention for reducing the risk of infection progression to disease and a key strategy in achieving tuberculosis elimination goals (5). However, due to several obstacles in the care of people eligible for preventive treatment of tuberculosis, less than 30.0% initiate treatment and less than 20.0% complete it (6). In Brazil, one of the potential barriers to the expansion of preventive treatment for tuberculosis is the limited number of lead trainers and health professionals certified to administer the tuberculin skin test, a crucial examination in evaluating latent tuberculosis infection in high-risk groups (7). 

An alternative test—the interferon gamma release assay (IGRA)—was incorporated into the Brazilian National Health System (*Sistema Único de Saúde*, SUS) only for priority populations, since it is less cost-effective than the tuberculin skin test (8). The World Health Organization (WHO) recommends the use of new skin tests, which use the same technique as the tuberculin skin test. Only 22.0% of the health centers in five Brazilian municipalities (Manaus, Rio de Janeiro, Porto Alegre, Recife, and São Paulo) with a high burden of tuberculosis offered tuberculin skin tests, and only 28.0% of these centers had qualified professionals to perform them (7). 

The tuberculin skin test is a simple examination that does not require laboratory infrastructure. It consists of the intradermal application of purified protein derivative (PPD), and between 48 and 96 hours after administration, the test is read to check for the presence of local induration; if present, its size is measured. The induration is a hardened, raised area that corresponds to the skin’s inflammatory response resulting from sensitization to the purified protein derivative and indicates possible prior exposure to the *Mycobacterium tuberculosis* (9). 

Requirements for professional certification for the tuberculin skin test vary between countries (10-13)although rates are now declining due to focused programs. The State of Indiana mandates continuous TB monitoring by health care workers who have completed an educational course. The Indiana State Department of Health (ISDH. Until December 2024, the Ministry of Health recommended that each professional to be trained perform between 80 and 100 tuberculin applications and the same number of induration readings (non-zero reactions), with a minimum agreement of 80.0% (reading difference between the trained professional’s measurement and that of the lead trainer within 2 mm), in double-blind readings (9). This training methodology has been empirically adopted by the Ministry of Health over the years, based on standardization studies—not training studies—from the 1960s. (14).

A simplified training protocol was sufficient to achieve high agreement in the readings of the tuberculin skin test, with no effect observed by the number of readings. However, this evaluation was limited to a small number of professionals in a single health center, due to the interruption of activities by the COVID-19 pandemic (15).

The low supply of the tuberculin test, the need for professional training, and the expected impact on the expansion of preventive tuberculosis treatment highlight the need to verify the effect of successive readings of the tuberculin skin test on the agreement of results during professional training (7). 

In the context of the expansion of preventive treatment in Brazil, this study, carried out under the ExpandTPT program, sought to identify the effect of successive readings of the tuberculin skin test on the proficiency of healthcare professionals using different indicators in five Brazilian cities: Manaus, Rio de Janeiro, Porto Alegre, Recife, and São Paulo. 

## Methods 

### Study design 

This was an operational observational study. This study aimed to verify the effect of successive tuberculin skin test readings on the proficiency of healthcare professionals using different indicators: agreement of results between the trained professional and the lead trainer, mean differences in readings, and sensitivity and specificity of the trained professional, considering the lead trainer as the gold standard. Data collection took place between October 2023 and June 2024. 

### Setting 

The study population consisted of healthcare professionals trained according to the official Ministry of Health protocol (9), with some simplifications for the application and reading of the tuberculin skin test, including the use of sausages and silicone models for practical simulation (15). The five included municipalities—Manaus, Rio de Janeiro, Porto Alegre, Recife, and São Paulo—were selected due to their high tuberculosis incidence burden, representation of different regions of the country, and having recently hosted training activities within the scope of the ExpandTPT program. 

The program, developed in partnership with the General Coordination for Tuberculosis Surveillance, Endemic Mycoses, and Nontuberculous Mycobacteria (*Coordenação-Geral de Vigilância da Tuberculose, Micoses Endêmicas e Micobactérias não Tuberculosas*, CGTM), the Brazilian Tuberculosis Research Network (*Rede Brasileira de Pesquisa em Tuberculose*, REDE-TB), and McGill University in Canada, began its activities in April 2023, intending to expand access to tuberculosis preventive treatment in Brazil. To this end, ExpandTPT promoted the training of healthcare professionals, incorporated innovative tools such as the simplified protocol for tuberculin skin test application and reading, implemented virtual quality control for test administration, and encouraged teams to identify barriers in contact management, proposing solutions adapted to the local context.

The tuberculin skin test trainings were conducted by experienced nurse trainers in the technique, recognized by the Ministry of Health as references for ensuring correct execution of the procedure, and therefore considered the gold standard. The process was carried out under national guidelines (9). 

Several complementary strategies were employed to overcome the challenge of reaching 100 arms for training. For tuberculin application, some trainees used sausages (15), and for reading induration, all used silicone models with varying indurations (16,17). An effort was made to schedule 100 tuberculin skin tests in each municipality; however, this target was not always achieved. The number of indurations was determined by the availability achieved during two application and reading shifts, and there was no requirement to reach a minimum number of readings. 

For induration measurement, caliper-type rulers (vernier calipers) were used, allowing for precise measurement in millimeters, as recommended for reading the tuberculin skin test (9). 

The measurements obtained during the reading of the tuberculin skin test, both by the professionals undergoing training and by the lead trainers, were recorded on individual forms known as reading maps (9). In the single map for each professional, the information was organized in chronological sequence, according to the order in which the readings were taken. 

After being completed, these reading maps underwent an anonymization process, during which all information that could identify who performed each reading was removed. 

### Participants 

All induration readings from tuberculin skin tests performed by both trained professionals and lead trainers were included. The trained professionals were previously selected and recommended by the municipal tuberculosis control programs and classified into two categories: with and without prior training.

### Variables 

The professional’s municipality of employment, the number of trained professionals, the number of readings performed, the tuberculin skin test results according to both the trained professional and the lead trainer, as well as the professional’s prior training status for administering the tuberculin test, were recorded. 

The results of the readings were analyzed by both frequency (absolute and relative) and categorization into three groups: 0-4 mm, 5-9 mm, and 10 mm or more. In Brazil, the tuberculin skin test is considered positive when the induration is ≥5 mm; however, in low-risk individuals, the cutoff for treatment indication is 10 mm (12). This explains the choice of cutoff points.

The differences between the readings of trained professionals and those of the lead trainers were calculated, with readings considered concordant when the difference was within 2 mm (9,13). The mean difference between the readings, as well as the concordance, sensitivity, and specificity, was calculated for each reading performed by the professionals. The results were organized in chronological order of the readings, from the 1st to the 50th. Not all professionals performed 50 readings, but all were included in the analysis, regardless of the number of readings performed, with censorship applied to the last one; that is, it was considered up to their last recorded reading. As the number of readings (professional-lead trainer) ranged from 168 for the first to 50 for the 50th reading, 50 readings were established as the acceptable threshold for inclusion in the analysis.

Throughout the readings, improvements of less than 5.0% were not considered substantial. For clinical decision-making (whether or not to prescribe preventive treatment for tuberculosis), sensitivity and specificity were more important than agreement around values that did not change the interpretation of the result from negative to positive and vice versa. The sensitivity and specificity of the trained professional’s reading were analyzed using the 5 mm and 10 mm cutoff values used in Brazil (18,19). 

### Data availability

The anonymized database used in this study is part of the ExpandTPT program, which, among other initiatives, promoted the training of healthcare professionals in application and reading of the tuberculin skin test. 

### Bias control

To avoid bias in the results, the forms were filled out in a blinded manner (trainers and lead trainers were unaware of each other’s readings). The database was anonymized, and the analyses were conducted by a researcher blinded to the participants’ variables. 

### Statistical methods

The statistical analysis was performed using the Statistical Package for the Social Sciences (IBM SPSS) software, version 21.0. The sensitivity and specificity of the readings performed by trained professionals were defined as the proportion of correctly identified positive and negative results, respectively, using the lead trainers as the gold standard. The agreement between readings of trained professionals and multipliers was assessed using the Kappa index, with values above 0.8 considered to indicate high agreement.

## Results 

There were 168 professionals: 108 in Manaus, 19 in Recife, 15 in São Paulo, 14 in Rio de Janeiro, and 12 in Porto Alegre. Of these, 90.0% were nurses; the others were nursing technicians or assistants. Seventeen (10.0%) reported prior training in tuberculin skin test administration and reading. Due to the small number of professionals with previous experience, except for Manaus, no comparative analyses were performed between the subgroups. The number of readings performed by each professional ranged from 5 to 122, with a mean of 35.3±29.2 readings (Table 1). The total number of readings was 5,929, of which 66.0% were positive (≥5 mm), with a slight concentration in the values of 10 mm and 15 mm. 

**Table 1:** Mean, standard deviation, minimum and maximum values, median, and interquartile range (IQR) of the number of tuberculin skin test readings, by municipality. Manaus, Porto Alegre, Recife, Rio de Janeiro, and São Paulo, 2023-2024 (n=168)

Municipality	n	Mean	Standard deviation	Minimum	Maximum	Median (interquartile range)
Manaus	108	16.4	6.0	5.0	35.0	14.0 (12.0-20.0)
**Porto Alegre**	12	53.7	15.2	10.0	66.0	55.0 (51.0-64.0)
Recife	19	53.1	16.1	28.0	72.0	58.0 (36.0-68.0)
**Rio de Janeiro**	14	91.2	6.9	75.0	98.0	93.5 (89.0-96.0)
**São Paulo**	15	82.3	19.5	67.0	122.0	76.0 (71.0-77.0)
Total	168	35.3	29.2	5.0	122.0	20.5 (14.0-62.5)

**Figure 1:**
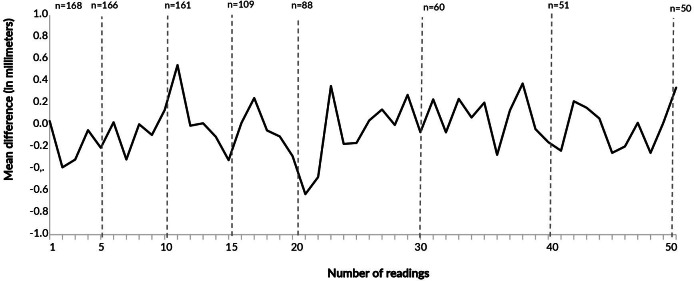
Number of professionals by number of tuberculin skin test readings performed, and mean difference (in millimeters) between readings by trained professionals and lead trainers, according to the reading order. Manaus, Porto Alegre, Recife, Rio de Janeiro, and São Paulo, 2023-2024 (n=168)

The mean difference between the readings of each of the 168 trained professionals and the lead trainers was 0.01 mm (±0,70). Considering only the first reading, the mean difference was 0.03 mm, and the differences remained stable throughout the readings, with no downward trend (Figure 1).

**Figure 2:**
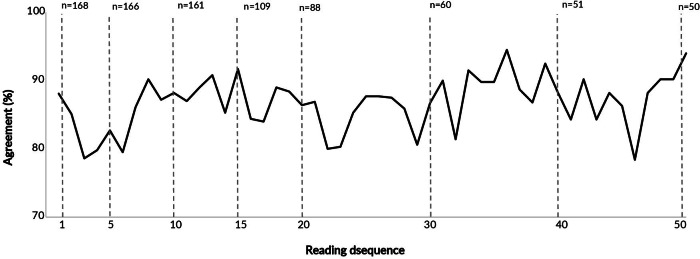
Number of professionals by number of tuberculin skin test readings performed, and percentage agreement between readings by trained professionals and lead trainers, according to the order of readings. Manaus, Porto Alegre, Recife, Rio de Janeiro, and São Paulo, 2023-2024 (n=168)

The agreement between the trained professionals and the multipliers was 93.0% for readings between 0 mm and 4 mm, 74.8% for readings between 5 mm and 9 mm, and 88.2% for readings of 10 mm or more (Kappa weighted 0.864) (Table 2). Agreement in the first reading was 88.1%, and no variations greater than 5.0% were observed (Figure 2). 

**Table 2:** Agreement between the tuberculin skin test readings by trained professionals and those by lead trainers, by municipality. Manaus, Porto Alegre, Recife, Rio de Janeiro, and São Paulo, 2023-2024 (n=168)

Municipality	Reading of professionals (millimeters)	Total	Reading of lead trainers (millimeters)			Weighted Kappa
			0-4	5-9	≥10	
		n (%)	n (%)	n (%)	n (%)	
Manaus	0-4	694 (39.3)	650 (94.9)	43 (11.7)	1 (0.1)	0.865
	5-9	401 (22.7)	35 (5.1)	271 (74.0)	95 (13.3)	
	≥10	671 (38.0)	0 (0.0)	52 (14.2)	619 (86.6)	
	Total	1,766 (100.0)	685 (100.0)	366 (100.0)	715 (100.0)	
**Porto Alegre**	0-4	169 (26.2)	152 (84.9)	16 (13.1)	1 (0.3)	0.828
	5-9	155 (24.1)	26 (14.5)	91 (74.6)	38 (11.1)	
	≥10	320 (49.7)	1 (0.6)	15 (12.3)	304 (88.6)	
	Total	644 (100.0)	179 (100.0)	122 (100.0)	343 (100.0)	
Recife	0-4	276 (27.4)	263 (95.3)	13 (6.8)	0 (0.0)	0.873
	5-9	193 (19.1)	13 (4.7)	135 (70.3)	45 (8.3)	
	≥10	539 (53.5)	0 (0.0)	44 (22.9)	495 (91.7)	
	Total	1,008 (100.0)	276 (100.0)	192 (100.0)	540 (100.0)	
**Rio de Janeiro**	0-4	343 (26.9)	310 (92.5)	30 (11.6)	3 (0.4)	0.847
	5-9	336 (26.3)	25 (7.5)	213 (82.6)	98 (14.3)	
	≥10	598 (46.8)	0 (0.0)	15 (5.8)	583 (85.2)	
	Total	1,277 (100.0)	335 (100.0)	258 (100.0)	684 (100.0)	
**São Paulo**	0-4	530 (42.9)	500 (92.3)	27 (15.3)	3 (0.6)	0.876
	5-9	207 (16.8)	39 (7.2)	123 (69.9)	45 (8.7)	
	≥10	497 (40.3)	3 (0.6)	26 (14.8)	468 (90.7)	
	Total	1,234 (100.0)	542 (100.0)	176 (100.0)	516 (100.0)	
Total	0-4	2,012 (33.9)	1,875 (93.0)	129 (11.6)	8 (0.3)	0.864
	5-9	1,292 (21.8)	138 (6.8)	833 (74.8)	321 (11.5)	
	≥10	2,625 (44.3)	4 (0.2)	152 (13.6)	2,469 (88.2)	
	Total	5,929 (100.0)	2,017 (100.0)	1,114 (100.0)	2,798 (100.0)	

**Figure 3:**
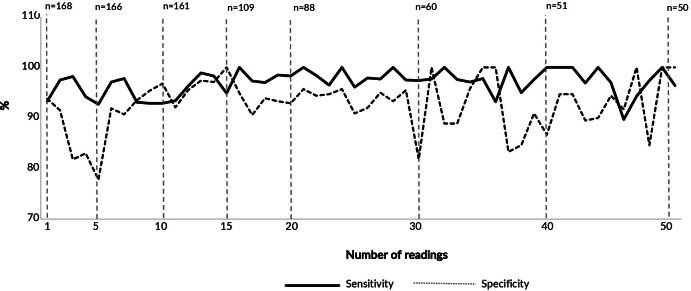
Number of professionals by number of tuberculin skin test readings performed and sensitivity and specificity estimates of trained professionals’ readings, considering lead trainers’ readings as the gold standard, for the 5 mm cutoff point, according to the number of readings. Manaus, Porto Alegre, Recife, Rio de Janeiro, and São Paulo, 2023-2024 (n=168)

The number of paired readings ranged from 168 professionals at the first to 50 professionals at the 50th reading. Considering the 5 mm cutoff, sensitivity and specificity were 96.5% and 93.0%, respectively, with values of 93.3% and 93.8% at the first reading. The values remained high in subsequent readings, with very small variations throughout, not exceeding 5.0% (Figure 3). For the 10 mm cutoff, sensitivity and specificity were 88.2% and 95.0%, respectively, with values of 93.0% and 96.3% at the first reading, again showing no substantive effect over successive readings. 

## Discussion 

In this study, no substantive or consistent effect of successive readings was observed on healthcare professionals’ proficiency in reading the tuberculin skin test, using multiple criteria: sensitivity and specificity (with the lead trainer’s reading as the gold standard), the mean difference between readings, and agreement between the trained professional and the lead trainer, considering a difference of up to 2 mm as acceptable. Since the first assessment, there was a high level of agreement between the induration readings of the tuberculin skin test obtained by trained professionals (with or without prior training) and those recorded by experienced lead trainers.

The mean difference between the readings of the two groups was small and remained stable throughout the execution of up to 50 readings. These findings suggest that the ability to measure induration does not show progressive improvement with an increasing number of readings, indicating consistent performance by the professionals from the beginning of the training process. 

Sensitivity was higher with the 5 mm cutoff, while specificity was slightly greater with the 10 mm cutoff. Regardless of the cutoff value, these metrics were high from the first reading and remained elevated, suggesting that no learning curve was observed over successive readings. Disagreements and decreases in accuracy occurred in a few readings, at random moments, not related to the chronological order. 

These findings are consistent with a previous small-scale pilot study, which also showed no influence of the number of readings on the agreement between trained professionals and lead trainers, even under a further simplified training protocol (15). No other studies or evidence were found in the literature regarding the number of arms required for training. This underscores the relevance of this study and future research comparing trainings with varying numbers of readings, or analyzing the effect of the number of readings on the trained professional’s skill.

The national recommendation in effect until December 2024, which required a minimum of 80 readings, is based on studies from the 1960s (9) regarding the standardization of the tuberculin skin test—rather than on training. This finding did not support that recommendation and contributed to the Ministry’s decision to revise the training protocol, requiring between seven and seventeen readings depending on the number of discrepancies (13). 

In most countries, although there is great variation in the requirements for professional certification in the application and reading of the tuberculin skin test, only a small number of supervised readings are required (between five and ten readings). International guidelines, such as those from the United States Centers for Disease Control and Prevention, the World Health Organization, and national institutions like the Public Health Agency of Canada, recognize that technical competence can be achieved with few supervised practices, provided they are conducted in controlled environments, following standardized protocols and under qualified supervision (11,12). This evidence reinforces the feasibility of streamlined and effective training models, especially in contexts with high demand for expanding access to tuberculin skin testing.

This study presents some limitations. The limited number of previously trained professionals included restricts the comparative analysis between readers with and without prior training. However, it is not expected that previously trained professionals will perform differently, nor is it expected that the number of readings will have an impact. Another limitation was the difficulty in reaching a total of 100 readings per professional, despite the efforts made. Nonetheless, the inclusion of all professionals, with sequential analysis of readings—from the first to the 50th, censoring at the last reading—allowed control of potential bias related to variation in the number of readings performed by each participant, since the number of readings did not impact the indicators used. The concentration of professionals in the same municipality was also limiting, which could generate bias if the skills or characteristics of these professionals influenced their aptitude. However, it is not expected that professionals from different municipalities possess different skills or have varying abilities to acquire them. 

A strength of this study was the inclusion of professionals from all geographic regions of Brazil, which allowed for some generalization of the findings. The double-blind reading of the induration and the blinded analysis of the anonymized database were additional strengths of the study. 

Longitudinal studies tracking professionals over time, assessing the impact of each reading on the improvement of tuberculin skin test interpretation, and validating tools as training instruments may further support the findings of this study.

No effect of successive readings on professionals’ proficiency was observed, indicating that technical competence in tuberculin skin test reading can be achieved with a reduced number of supervised practices following properly structured training. These results, together with other evidence, supported the revision of the national training guidelines, contributing to the adoption of more streamlined and efficient training models. 

## References

[1] Trajman A, Campbell JR, Kunor T, Ruslami R, Amanullah F, Behr MA (2025). Tuberculosis. The Lancet.

[2] World Health Organization (2024). Global Tuberculosis Report 2024 [Internet].

[3] Houben RMGJ, Dodd PJ (2016). The Global Burden of Latent Tuberculosis Infection: A Re-estimation Using Mathematical Modelling.

[4] Dowdy DW, Behr MA (2022). Are we underestimating the annual risk of infection with Mycobacterium tuberculosis in high-burden settings? Lancet Infect Dis [Internet].

[5] World Health Organization (2023). Global tuberculosis report 2023 [Internet].

[6] Alsdurf H, Hill PC, Matteelli A, Getahun H, Menzies D (2016). The cascade of care in diagnosis and treatment of latent tuberculosis infection: a systematic review and meta-analysis. Lancet Infect Dis.

[7] Cordeiro DC, Kuabara P, Amaral BC, Portugal LM, Sacramento DS, Oliveira LR de (2025). Needs assessment and preparedness of the primary health care network for scaling-up preventive tuberculosis treatment in 5 Brazilian capitals.

[8] Steffen RE, Caetano R, Pinto M, Chaves D, Ferrari R, Bastos M (2013). Cost-Effectiveness of Quantiferon®-TB Gold-In-Tube Versus Tuberculin Skin Testing for Contact Screening and Treatment of Latent Tuberculosis Infection in Brazil.

[9] Ministério da Saúde (2014). Técnicas de aplicação e leitura de prova tuberculínica.

[10] Stone CL (2001). Building academic and practical knowledge in nursing through TB skin testing certification in a BSN curriculum. Public Health Nurs Boston Mass.

[11] (2025). Canadian-Tuberculosis-Standards_7th-edition_Complete.

[12] Centers for Disease Control and Prevention (2025). Tuberculosis (TB).

[13] Ministério das Saúde (2024). Nota informativa CGTM/.DATHI/SVSA/MS No 14 de 03 de dezembro de 2024. Dispõe sobre atualização das recomendações da capacitação dos profissionais de saúde nas técnicas de aplicação e leitura da Prova Tuberculínica (PT) no SUS.

[14] Teruel JR, Ruffino Netto A, Duarte GG (1969). Standardization of tuberculin test readers.

[15] Gloria LL, Bastos ML, Santos JB, Trajman A (2021). A simple protocol for tuberculin skin test reading certification. Cad Saúde Pública.

[16] (2024). Repositório ExpandTPT.

[17] (2024). Web I. Trabalhos - 59^o^ MEDTROP - Congresso Brasileiro da Sociedade Brasileira de Medicina Tropical [Internet].

[18] Ministério da Saúde (BR) (2019). Manual de Recomendações para o Controle da Tuberculose no Brasil [Internet].

[19] Ministério da Saúde (BR) (2022). Protocolo de vigilância da infecção latente pelo Mycobacterium tuberculosis no Brasil.

