# Supplementary material for: Effect of successive tuberculin skin test readings on healthcare professionals’ proficiency: an operational observational study in five high-tuberculosis-burden Brazilian municipalities, 2023-2024
Source: Epidemiol Serv Saude. 2025 Oct 31;34:e20240795. doi: 10.1590/S2237-96222025v34e20240795.en (PMC12582531; doi:10.1590/S2237-96222025v34e20240795.en)
Supplement: Translation

# Translation: Efeito de sucessivas leituras da prova tuberculínica na aptidão dos profissionais de saúde: estudo observacional operacional em cinco municípios brasileiros de alta carga da tuberculose, 2023-2024

**DOI:** 10.1590/S2237-96222025v34e20240795.pt

**Authors:** Dinah Carvalho Cordeiro, Elisa Barroso de Aguiar, Ronir Raggio Luiz, Daniel Souza Sacramento, Priscilla Lúcia Wolter Paolino, Maria Jacirema Ferreira Gonçalves, Anete Trajman

**Affiliations:** 1 Universidade Federal do Rio de Janeiro, Programa de Pós-Graduação em Clínica Médica, Rio de Janeiro, RJ, Brasil; 2 Universidade Federal do Rio de Janeiro, Instituto de Estudos em Saúde Coletiva, Rio de Janeiro, RJ, Brasil; 3 Secretaria Municipal de Saúde de Manaus, Departamento de Vigilância Epidemiológica, Ambiental, Zoonoses e da Saúde do Trabalhador, Manaus, AM, Brasil; 4 Rede Brasileira de Pesquisa em Tuberculose, Rio de Janeiro, RJ, Brasil; 5 Universidade Federal do Amazonas, Programa de Pós-Graduação em Enfermagem, Manaus, AM, Brasil

**Keywords:** Infecção Latente, Cursos de Capacitação, Teste Tuberculínico, Serviços de Saúde, Estudo Observacional

## Abstract

**Objetivo:**

Verificar o efeito de sucessivas leituras da prova tuberculínica na aptidão dos profissionais de saúde.

**Métodos:**

Tratou-se de estudo observacional operacional que comparou as leituras de profissionais e multiplicadores em capacitações conduzidas pelo Ministério da Saúde em cinco municípios brasileiros. Calculou-se a diferença média entre as leituras, a concordância, a sensibilidade e a especificidade considerando o multiplicador como padrão-ouro em cada uma das primeiras 50 leituras. A concordância interobservador foi avaliada pelo índice Kappa. O banco de dados foi oriundo de um programa de capacitação em leitura da prova tuberculínica. Utilizou-se o software Statistical Package for the Social Sciences Statistics para as análises descritivas.

**Resultados:**

Capacitaram-se 168 profissionais e houve 5.929 leituras. O número médio de leituras por profissional foi 35,3±29,2 (mínimo=5, máximo=122). A diferença média entre as leituras dos profissionais e as dos multiplicadores foi 0,01 mm (±0,70 mm). A concordância foi 93,0% para leituras entre 0 mm e 4 mm, 74,8% para leituras entre 5 mm e 9 mm e 88,2% para leituras de 10 mm ou mais (Kappa ponderado 0,864). A sensibilidade e a especificidade foram 96,5% e 93,0% para o ponto de corte de 5 mm (mais usado no Brasil) e 88,2% e 95,0% para 10 mm. Nenhum dos parâmetros avaliados sofreu alteração ao longo das 50 primeiras leituras.

**Conclusão:**

Não houve efeito de leituras sucessivas na habilidade dos profissionais, que foi elevada desde a primeira leitura de acordo com todos os parâmetros. Esses achados corroboram as novas recomendações de capacitação do Ministério da Saúde recém-publicadas.

**Aspectos éticos**

Esta pesquisa respeitou os princípios éticos, obtendo os seguintes dados de aprovação:

Comitê de ética em pesquisa: Universidade Federal do Rio de Janeiro

Número do parecer: 5.936.814

Data de aprovação: 10/3/2023

Certificado de apresentação de apreciação ética: 67116923.3.0000.5257

Registro do consentimento livre e esclarecido: Dispensado.

## Introdução 

Causada pelo agente infeccioso *Mycobacterium tuberculosis*, a tuberculose afeta predominantemente os pulmões e é transmitida por via aérea. A transmissão requer contato próximo e prolongado e ocorre quando indivíduos com tuberculose pulmonar e não tratada eliminam aerossóis que contêm bacilos ao tossir, espirrar, cantar ou falar (1).

Em 2023, mais de 10 milhões de pessoas adoeceram de tuberculose no mundo, com a doença reassumindo a posição de principal causa de morte por um único agente infeccioso naquele ano (2). 

Em 2011, estimou-se que 23,0% da população mundial estaria infectada por *Mycobacterium tuberculosis* (3). Nessa condição, a bactéria permanece viável no organismo de forma inativa, sem causar sintomas e sem risco de transmissão. No entanto, aproximadamente 5,0% a 10,0% desses indivíduos podem desenvolver a doença ao longo da vida, enquanto outros conseguirão eliminar a infecção naturalmente (4). A maior parte dos novos diagnósticos de tuberculose advém desse contingente de quase 2 bilhões de pessoas com a infecção (1). 

Após a infecção inicial, o risco de progressão para a tuberculose é influenciado por diversos fatores, incluindo o estado imune e as condições de saúde associadas. Os sintomas clássicos incluem tosse persistente, dor torácica, fadiga, perda de peso, febre e sudorese noturna (1).

O tratamento preventivo da tuberculose é a principal intervenção para a redução do risco de progressão da infecção para doença e uma estratégia fundamental nas metas de eliminação da tuberculose (5). Entretanto, devido às várias barreiras no cuidado das pessoas elegíveis para o tratamento preventivo da tuberculose, menos de 30,0% iniciam o tratamento e menos de 20,0% o completam (6). No Brasil, uma das possíveis barreiras à ampliação do tratamento preventivo da tuberculose é o reduzido número de capacitadores e de profissionais de saúde certificados para realização da prova tuberculínica, exame fundamental na avaliação da infecção latente por tuberculose em grupos de risco (7). 

Um exame alternativo – o ensaio de liberação do interferon gama – foi incorporado ao Sistema Único de Saúde apenas em populações prioritárias, visto que é menos custo efetivo do que a prova tuberculínica (8). A Organização Mundial da Saúde recomenda o uso de novos testes cutâneos, os quais utilizam a mesma técnica que a prova tuberculínica. Apenas 22,0% das unidades básicas de cinco municípios brasileiros (Manaus, Rio de Janeiro, Porto Alegre, Recife e São Paulo) com alta carga de tuberculose ofereciam prova tuberculínica, e somente 28,0% dessas unidades tinham profissionais qualificados para a realização desse exame (7). 

A prova tuberculínica é um exame simples que dispensa infraestrutura laboratorial. Consiste na aplicação intradérmica do derivado proteico purificado e, entre 48 e 96 horas da aplicação, realiza-se a leitura para verificar se há presença de endurado local, e, se houver, mede-se o tamanho. O endurado é uma elevação endurecida que corresponde à resposta inflamatória da pele, resultante da sensibilização do organismo ao derivado proteico purificado, e indica possível exposição prévia ao *Mycobacterium tuberculosis* (9). 

As exigências para a certificação profissional para prova tuberculínica variam entre os países (10-13). O Ministério da Saúde recomendava, até dezembro de 2024, que cada profissional a ser capacitado realizasse entre 80 e 100 aplicações de tuberculina e o mesmo número de leituras do endurado (reações diferentes de zero), com no mínimo 80,0% de concordância (diferença de leitura entre a medida do profissional capacitado e a do multiplicador de até 2 mm), em leituras duplo-cegas (9). Essa metodologia de capacitação foi assumida empiricamente ao longo dos anos pelo Ministério da Saúde, com base em estudos de padronização – e não de capacitação – da década de 1960 (14).

Um protocolo simplificado de capacitação foi suficiente para alcançar a alta concordância nas leituras da prova tuberculínica, sem efeito observado pelo número de leituras. Entretanto, esta avaliação foi limitada ao pequeno número de profissionais em uma única unidade de saúde, devido à interrupção das atividades pela pandemia de covid-19 (15).

A baixa oferta da prova tuberculínica, a necessidade de capacitação de profissionais e o impacto esperado na ampliação do tratamento preventivo da tuberculose evidenciam a necessidade de verificar o efeito das sucessivas leituras da prova tuberculínica na concordância dos resultados durante a capacitação de profissionais (7). 

No contexto da expansão do tratamento preventivo no Brasil, este estudo, realizado no âmbito do programa ExpandTPT, buscou verificar o efeito de sucessivas leituras da prova tuberculínica na aptidão dos profissionais de saúde utilizando diferentes indicadores em cinco municípios brasileiros: Manaus, Rio de Janeiro, Porto Alegre, Recife e São Paulo. 

## Métodos 

### Delineamento do estudo 

Tratou-se de estudo observacional operacional. Visou-se verificar o efeito de sucessivas leituras da prova tuberculínica na aptidão dos profissionais de saúde utilizando diferentes indicadores: concordância dos resultados entre capacitado e multiplicador, média das diferenças das leituras e sensibilidade e especificidade do capacitado, considerando o multiplicador como padrão-ouro. A coleta de dados ocorreu entre outubro de 2023 e junho de 2024. 

### Contexto 

A população do estudo consistiu em profissionais de saúde capacitados com o protocolo oficial do Ministério da Saúde (9), com algumas simplificações para a aplicação e leitura da prova tuberculínica, incluindo o uso de salsichas e modelos em silicone para simulação prática (15). Os cinco municípios incluídos – Manaus, Rio de Janeiro, Porto Alegre, Recife e São Paulo – foram selecionados por apresentarem alta carga de incidência de tuberculose, representarem diferentes regiões do país e terem sido local de capacitação recente no âmbito do programa ExpandTPT. 

O programa, desenvolvido em parceria com a Coordenação-Geral de Vigilância da Tuberculose, Micoses Endêmicas e Micobactérias não Tuberculosas, a Rede Brasileira de Pesquisas em Tuberculose e a Universidade McGill, no Canadá, iniciou suas atividades em abril de 2023 com o objetivo de ampliar o acesso ao tratamento preventivo da tuberculose no Brasil. Para isso, o ExpandTPT promoveu a capacitação de profissionais de saúde, incorporou ferramentas inovadoras, como o protocolo simplificado para aplicação e leitura da prova tuberculínica, implementou o controle de qualidade virtual da aplicação da prova tuberculínica e estimulou as equipes a identificar empecilhos nos cuidados dos contatos, propondo soluções adaptadas à realidade local.

As capacitações da prova tuberculínica foram conduzidas por enfermeiras multiplicadoras experientes na aplicação da técnica da prova tuberculínica, consideradas referências pelo Ministério da Saúde por assegurar a execução correta do procedimento, e por isso foram consideradas padrão-ouro. Todo o processo foi realizado em conformidade com as recomendações nacionais (9). 

Algumas estratégias complementares foram utilizadas para superar a dificuldade de alcançar o número de 100 braços para a realização da capacitação: para a aplicação da tuberculina, alguns profissionais em capacitação utilizaram salsichas (15), e, para a leitura do endurado, todos utilizaram modelos em silicone com diferentes tamanhos de endurados (16,17). Um esforço foi feito para o agendamento de 100 provas tuberculínicas em cada município, mas esse número nem sempre foi alcançado. O número de endurados foi estabelecido pela disponibilidade alcançada em dois turnos de aplicação e leitura, não sendo obrigatório alcançar um número mínimo de leituras. 

Para a medição do endurado, foram utilizadas réguas do tipo caliper (paquímetro), que permitiram a mensuração precisa em milímetros, conforme recomendado para a leitura da prova tuberculínica (9). 

As medidas obtidas durante a leitura da prova tuberculínica, tanto pelos profissionais que estavam sendo capacitados quanto pelos multiplicadores, foram anotadas em formulários individuais chamados de mapas de leitura (9). No mapa único de cada profissional, as informações eram organizadas de acordo com a ordem em que as leituras foram feitas, ou seja, seguindo a sequência cronológica. 

Depois de preenchidos, esses mapas passaram pelo processo de anonimização, no qual foram retiradas todas as informações que pudessem identificar quem fez cada leitura. 

### Participantes 

Foram incluídas todas as leituras dos endurados das provas tuberculínicas realizadas pelos profissionais capacitados e multiplicadores. Os profissionais capacitados foram previamente selecionados e indicados pelos programas municipais de controle da tuberculose, e classificados em duas categorias: com e sem capacitação prévia.

### Variáveis 

O munícipio de trabalho do profissional, o número de profissionais capacitados, o número de leituras realizadas, o resultado da prova tuberculínica de acordo com o profissional capacitado e com o multiplicador, além do profissional com/sem capacitação na realização da prova tuberculínica, foram registrados. 

Os resultados das leituras foram analisados pela frequência (absoluta e relativa) e pela categorização 0-4 mm, 5-9 mm e 10 mm ou mais. No Brasil, a prova tuberculínica é considerada positiva quando o endurado é ≥5 mm, mas, em pessoas de menor risco, o ponto de corte para a indicação do tratamento é 10 mm (12), por isso as escolhas de pontos de corte.

As diferenças entre as leituras dos profissionais capacitados e as dos multiplicadores foram calculadas, sendo consideradas concordantes as leituras com diferença de até 2 mm (9,13). A média das diferenças entre as leituras, bem como a concordância, a sensibilidade e a especificidade, foi calculada em cada uma das leituras realizadas pelos profissionais. Os resultados foram organizados na ordem cronológica da realização das leituras, da primeira à 50ª. Nem todos os profissionais realizaram 50 leituras, mas todos foram incluídos na análise, independentemente do número de leituras realizadas, com censura na última, ou seja, considerou-se até sua última leitura registrada. Como o número de leituras (profissional-multiplicador) variou de 168 para a primeira até 50 para 50ª leitura, estabeleceram-se 50 leituras como o limite aceitável para incluir na análise.

Ao longo das leituras, melhorias de menos de 5,0% não foram consideradas substantivas. Para a tomada de decisão clínica (prescrever ou não o tratamento preventivo da tuberculose), a sensibilidade e a especificidade foram mais importantes do que a concordância em torno de valores que não mudaram a interpretação do resultado de negativo para positivo e vice-versa. Foram analisadas a sensibilidade e a especificidade da leitura do capacitado utilizando os valores de corte de 5 mm e 10 mm – utilizados no Brasil (18,19). 

### Fonte de dados

O banco de dados anonimizado utilizado neste estudo faz parte do programa ExpandTPT, que, entre outras ações, promoveu a capacitação de profissionais de saúde na aplicação e leitura da prova tuberculínica. 

### Controle de viés

Para evitar vieses nos resultados, os mapas foram preenchidos de forma cega (capacitadores e multiplicadores não estavam cientes das leituras mútuas). O banco foi anonimizado, e as análises foram realizadas por pesquisador cego para as variáveis dos participantes. 

### Métodos estatísticos

A análise estatística foi realizada no software Statistical Package for the Social Sciences (IBM SPSS) Statistics, versão 21.0. A sensibilidade e a especificidade da leitura do profissional capacitado foram definidas como a proporção de leituras corretamente avaliadas como positivas e negativas, respectivamente, considerando o multiplicador como padrão-ouro. A concordância entre as leituras dos profissionais capacitados e as dos multiplicadores foi realizada por meio do índice Kappa, cujo valor acima de 0,8 foi considerado de alta concordância.

## Resultados 

Foram capacitados 168 profissionais: 108 em Manaus, 19 em Recife, 15 em São Paulo, 14 no Rio de Janeiro e 12 em Porto Alegre. Destes, 90,0% eram enfermeiros; os demais, técnicos ou auxiliares de enfermagem. Dezessete ( ,0%) relataram capacitação prévia para aplicação e leitura da prova tuberculínica. Devido ao pequeno número de profissionais com experiência prévia, exceto Manaus, não foram feitas análises comparativas entre os subgrupos. A quantidade de leituras realizadas por cada profissional variou de 5 a 122, com média de 35,3±29,2 leituras (Tabela 1). O total de leituras foi 5.929, das quais 66,0% eram positivas (≥5 mm), com discreta concentração nos valores de 10 mm e 15 mm. 

**Tabela 1:** Média, desvio-padrão, valores mínimo e máximo, mediana e intervalo interquartil do número de leituras da prova tuberculínica segundo o município. Manaus, Porto Alegre, Recife, Rio de Janeiro e São Paulo, 2023-2024 (n=168)

Município	n	Média	Desvio-padrão	Mínimo	Máximo	Mediana (intervalo interquartil)
Manaus	108	16,4	6,0	5,0	35,0	14,0 (12,0-20,0)
**Porto Alegre**	12	53,7	15,2	10,0	66,0	55,0 (51,0-64,0)
Recife	19	53,1	16,1	28,0	72,0	58,0 (36,0-68,0)
**Rio de Janeiro**	14	91,2	6,9	75,0	98,0	93,5 (89,0-96,0)
**São Paulo**	15	82,3	19,5	67,0	122,0	76,0 (71,0-77,0)
Total	168	35,3	29,2	5,0	122,0	20,5 (14,0-62,5)

A média das diferenças das leituras de cada um dos 168 profissionais capacitados em relação aos multiplicadores foi 0,01 mm (±0,70). Considerando apenas a primeira leitura, a diferença média foi 0,03 mm, e as diferenças permaneceram estáveis ao longo das leituras, sem tendência de redução (Figura 1).

**Figura 1:** Número de profissionais conforme número de leituras realizadas da prova tuberculínica, e diferença média, em milímetros, entre as leituras dos profissionais capacitados e as dos multiplicadores, segundo a ordem de leituras. Manaus, Porto Alegre, Recife, Rio de Janeiro e São Paulo, 2023-2024 (n=168)

A concordância entre os profissionais capacitados e os multiplicadores foi 93,0% para leituras entre 0 mm e 4 mm, 74,8% para leituras entre 5 mm e 9 mm e 88,2% para 10 mm ou mais (Kappa ponderado 0,864) (Tabela 2). A concordância na primeira leitura foi 88,1%, e não se observaram variações superiores a 5,0% (Figura 2). 

**Tabela 2:** Concordância entre a leitura da prova tuberculínica dos profissionais capacitados, segundo a leitura dos multiplicadores, por município. Manaus, Porto Alegre, Recife, Rio de Janeiro e São Paulo, 2023-2024 (n=168)

Município	Leitura dos profissionais (milímetros)	Total	Leitura do multiplicador (milímetros)			Kappa ponderado
			0-4	5-9	≥10	
		n (%)	n (%)	n (%)	n (%)	
Manaus	0-4	694 (39,3)	650 (94,9)	43 (11,7)	1 (0,1)	0,865
	5-9	401 (22,7)	35 (5,1)	271 (74,0)	95 (13,3)	
	≥10	671 (38,0)	0 (0,0)	52 (14,2)	619 (86,6)	
	Total	1.766 (100,0)	685 (100,0)	366 (100,0)	715 (100,0)	
**Porto Alegre**	0-4	169 (26,2)	152 (84,9)	16 (13,1)	1 (0,3)	0,828
	5-9	155 (24,1)	26 (14,5)	91 (74,6)	38 (11,1)	
	≥10	320 49,7)	1 (0,6)	15 (12,3)	304 (88,6)	
	Total	644 (100,0)	179 (100,0)	122 (100,0)	343 (100,0)	
Recife	0-4	276 (27,4)	263 (95,3)	13 (6,8)	0 (0,0)	0,873
	5-9	193 (19,1)	13 (4,7)	135 (70,3)	45 (8,3)	
	≥10	539 (53,5	0 (0,0)	44 (22,9)	495 (91,7)	
	Total	1.008 (100,0)	276 (100,0)	192 (100,0)	540 (100,0)	
**Rio de Janeiro**	0-4	343 (26,9)	310 (92,5)	30 (11,6)	3 (0,4)	0,847
	5-9	336 (26,3)	25 (7,5)	213 (82,6)	98 (14,3)	
	≥10	598 (46,8)	0 (0,0)	15 (5,8)	583 (85,2)	
	Total	1.277 (100,0)	335 (100,0)	258 (100,0)	684 (100,0)	
**São Paulo**	0-4	530 (42,9)	500 (92,3)	27 (15,3)	3 (0,6)	0,876
	5-9	207 (16,8)	39 (7,2)	123 (69,9)	45 (8,7)	
	≥10	497 (40,3)	3 (0,6)	26 (14,8)	468 (90,7)	
	Total	1.234 (100,0)	542 (100,0)	176 (100,0)	516 (100,0)	
Total	0-4	2.012 (33,9)	1.875 (93,0)	129 (11,6	8 (0,3)	0,864
	5-9	1.292 (21,8)	138 (6,8)	833 (74,8)	321 (11,5)	
	≥10	2.625 (44,3)	4 (0,2)	152 (13,6)	2.469 (88,2)	
	Total	5.929 (100,0)	2.017 (100,0)	1.114 (100,0)	2.798 (100,0)	

**Figura 2:** Número de profissionais conforme número de leituras realizadas da prova tuberculínica e percentual de concordância entre as leituras dos profissionais capacitados e as dos multiplicadores, segundo a ordem de leituras. Manaus, Porto Alegre, Recife, Rio de Janeiro e São Paulo, 2023-2024 (n=168)

O número de leituras pareadas variou de 168 profissionais para a primeira até 50 profissionais para a 50ª leitura. Considerando o ponto de corte de 5 mm, a sensibilidade e a especificidade foram 96,5% e 93,0%, sendo de 93,3% e 93,8% na primeira leitura. Os valores permaneceram elevados nas leituras subsequentes, com variações muito pequenas ao longo das leituras, que não ultrapassam 5,0% (Figura 3). Para o valor de corte de 10 mm, a sensibilidade e a especificidade foram 88,2% e 95,0%, sendo 93,0% e 96,3% na primeira leitura, novamente sem efeito substantivo ao longo das leituras sucessivas. 

**Figura 3:** Número de profissionais conforme número de leituras realizadas da prova tuberculínica e estimativas de sensibilidade e especificidade das leituras dos profissionais capacitados, considerando as leituras dos multiplicadores como padrão-ouro, para o ponto de corte de 5 mm, segundo o número de leituras. Manaus, Porto Alegre, Recife, Rio de Janeiro e São Paulo, 2023-2024 (n=168)

## Discussão 

Neste estudo, observou-se que sucessivas leituras não têm efeito substantivo ou consistente na aptidão para leitura da prova tuberculínica do profissional, utilizando diferentes critérios: sensibilidade e especificidade (considerando a leitura do multiplicador como padrão-ouro), diferença média das leituras e concordância entre profissional capacitado e multiplicador, considerando até 2 mm de diferença aceitável. Desde a primeira avaliação, houve elevada concordância entre as medidas de endurado da prova tuberculínica obtidas por profissionais capacitados (com ou sem capacitação prévia) e aquelas registradas por multiplicadores experientes.

A diferença média entre as leituras dos dois grupos foi pequena e manteve-se estável ao longo da execução de até 50 leituras. Esses resultados sugerem que a habilidade de mensuração do endurado não apresenta melhora progressiva relacionada ao aumento do número de leituras, o que indica desempenho consistente dos profissionais desde o início do processo de capacitação. 

A sensibilidade foi superior com o valor de 5 mm e a especificidade foi discretamente maior com o valor de 10 mm. Independentemente do valor de corte, esses valores foram elevados desde a primeira leitura e permaneceram altos, o que sugeriu que não houve curva de aprendizagem ao longo das leituras sucessivas. Discordâncias e queda de acurácia aconteceram em poucas leituras, em momentos aleatórios, não relacionadas à ordem cronológica. 

Estes resultados corroboram um pequeno estudo piloto prévio, em que não houve influência do número de leituras na concordância entre capacitados e multiplicadores com capacitação ainda mais simplificada (15). Não foram encontrados na literatura outros estudos e evidências sobre o número de braços necessários para a capacitação. Isso demonstra a relevância deste estudo e de outros que venham a comparar capacitações utilizando diferentes números de leituras, ou que analisem o efeito do número de leituras na habilidade do profissional capacitado 

A recomendação nacional vigente até dezembro de 2024, que exigia o número mínimo de 80 leituras concordantes, deriva de estudos sobre a padronização da prova tuberculínica da década de 1960 (9) – e não sobre a capacitação. Este achado não sustentou essa recomendação e contribuiu para a decisão ministerial de mudar a capacitação, com exigência de 7 a 17 leituras, a depender do número de discordâncias (13). 

Na maior parte dos países, embora haja grande variação nas exigências para a certificação do profissional na aplicação e leitura da prova tuberculínica, apenas um pequeno número de leituras supervisionadas é exigido (entre cinco e dez leituras). Diretrizes internacionais, como as do Centers for Disease Control and Prevention dos Estados Unidos, as da Organização Mundial da Saúde e as de órgãos nacionais como o Public Health Agency of Canada, reconhecem que a competência técnica pode ser alcançada com poucas práticas supervisionadas, desde que conduzidas em ambiente controlado, com protocolos padronizados e supervisão qualificada (11,12). Essas evidências reforçam a viabilidade de modelos de capacitação mais enxutos e eficazes, especialmente em contextos de alta demanda por ampliação do acesso ao teste tuberculínico.

Este estudo tem limitações. O número limitado de profissionais previamente capacitados incluídos restringe a análise comparativa entre leitores com capacitação prévia ou não. No entanto, não é esperado que profissionais previamente capacitados tenham desempenho diferente ou que o número de leituras tenha impacto. Outra limitação foi a dificuldade em atingir o total de 100 leituras por profissional, apesar dos esforços realizados. Ainda assim, a inclusão de todos os profissionais, com análise sequencial das leituras – da primeira à 50ª, com censura na última leitura – permitiu controlar o viés potencial relacionado à variação no número de leituras executadas por cada participante, uma vez que o número de leituras não impacta os indicadores utilizados. Foi limitante também a concentração dos profissionais em um mesmo município, o que poderia gerar viés caso as habilidades ou características desses profissionais influenciassem sua aptidão. Todavia, não é esperado que profissionais de diferentes municípios tenham habilidades diferentes ou que tenham capacidade de adquirir habilidade de forma diferente. 

Uma potencialidade deste estudo foi a inclusão de profissionais de todas as regiões geográficas do Brasil, o que permitiu certa generalização dos achados. A leitura duplo-cega do endurado e a análise cega em banco anonimizado foram outras potencialidades. 

Estudos que acompanhem os profissionais a longo prazo, acompanhem o impacto de cada leitura na melhoria da leitura da prova tuberculínica e validem ferramentas como instrumento de capacitação podem corroborar os achados deste estudo.

Não houve efeito de leituras sucessivas na habilidade dos profissionais, e isso indica que a competência técnica para leitura da prova tuberculínica pode ser alcançada com o número reduzido de práticas supervisionadas, a partir da capacitação adequadamente estruturada. Esses resultados fundamentaram, juntamente com outras evidências, a revisão da recomendação nacional de capacitação, o que contribui para a adoção de modelos mais ágeis e eficientes.
